# Salt Tolerance of Rice Is Enhanced by the *SS3* Gene, Which Regulates Ascorbic Acid Synthesis and ROS Scavenging

**DOI:** 10.3390/ijms231810338

**Published:** 2022-09-07

**Authors:** Guang Chen, Huimin Han, Xiuli Yang, Ruiying Du, Xu Wang

**Affiliations:** 1 Institute of Quality Standard and Monitoring Technology for Agro-Products of Guangdong Academy of Agricultural Sciences, Guangzhou 510640, China; 2 Key Laboratory of Testing and Evaluation for Agro-Product Safety and Quality, Ministry of Agriculture and Rural Affairs, Guangzhou 510640, China; 3 Guangdong Provincial Key Laboratory of Quality & Safety Risk Assessment for Agro-Products, Guangzhou 510640, China; 4 State Key Laboratory of Cotton Biology, Institute of Cotton Research of Chinese Academy of Agricultural Sciences, Anyang 455000, China

**Keywords:** rice, salt stress, mannose-1-phosphate guanylyltransferase, ascorbic acid, reactive oxygen species

## Abstract

Mining the key genes involved in the balance of rice salt tolerance is extremely important for developing salt-tolerant rice varieties. A library of *japonica* mutants was screened under salinity conditions to identify putative salt stress-responsive genes. We identified a highly salt-sensitive mutant *ss3* and used a map-based cloning approach to isolate the gene *SS3*, which encodes mannose-1-phosphate guanylyltransferase. Under salt treatment, *ss3* mutants have decreased ascorbic acid (AsA) content and increased reactive oxygen species (ROS) levels compared with the wild type (WT). Exogenous AsA restored the salt tolerance of *ss3* plants, indicating that inhibition of AsA synthesis was an important factor in the salt sensitivity of the mutant. Functional complementation using the WT allele rescued the mutation, and transcription of *SS3* was induced by salt stress. Vector *SS3p:SS3* was constructed containing the 1086 bp coding sequence of *SS3*. Under salinity conditions, transgenic seedlings expressing *SS3p:SS3* had improved salt tolerance relative to WT, as demonstrated by better growth status, higher chlorophyll content, a lower level of Na^+^, and a reduced Na^+^/K^+^ ratio. Further investigation revealed that several senescence- and autophagy-related genes were expressed at lower levels in salt-stressed transgenic lines compared to WT. These results demonstrate the positive impact of *SS3* on salt tolerance in rice through the regulation of AsA synthesis and ROS accumulation, and indicate that *SS3* is a valuable target for genetic manipulation.

## 1. Introduction

Rice is a dietary staple for much of the world’s population. To ensure food security, it is essential to increase production by utilizing marginal land including extensive saline areas where the yields of current elite varieties are reduced [1]. In the presence of salt, growth is inhibited throughout development [2] but particularly at the early seedling stage [3]. Normal progression of rice plants here is a strong indicator of overall tolerance to salinity.

Plants grown under salt stress exhibit many adverse effects. In particular, damage to membranes can lead to lipid peroxidation and increased formation of ROS above normal homeostasis levels [4] with the concomitant accumulation of malondialdehyde (MDA) [5], which may further disrupt the fundamental components of the cell. Thus, plants have developed mechanisms to control undesirable oxidation, including antioxidant enzymes and low-molecular-weight compounds such as carotenoids, glutathione, flavonoids, and ascorbic acid (AsA) [6].

AsA is a ubiquitous scavenger of ROS [7] and, in tomatoes, increased levels have been associated with resistance to salt [8]. Its synthesis in higher plants is controlled by the D-mannose/L-galactose pathway, all the components of which have been characterized [9,10]. The importance of the L-galactose pathway in AsA synthesis has been demonstrated by studying multiple mutants. In *Arabidopsis*, the *VTC2* gene encodes a GDP-L-galactose phosphorylase. The AsA content in *vtc2-1* and *vtc2-2* mutants is only ~20% of that in the wild type and is sensitive to ozone [11]. In the T-DNA knockout *vtc4* (L-galactose-1-phosphate phosphatase) *Arabidopsis* mutant, AsA content and seed germination rate decreased [12]. *vtc1* is a knock-down mutant of mannose-1-phosphate guanylate transferase (GDP-mannose pyrophosphorylase, EC 2.7.7.13, MPGase) gene that to contains only 25% of the AsA level compared with the wild type [13]. *OsVTC1* in the rice genome consists of three homologous genes. Among these homologs, *OsVTC1-1* is closely related to the formation of AsA in rice leaves, while *OsVTC1-3* plays an important role in the synthesis of AsA in roots [14]. Enhancing *VTC1* expression increases the production of AsA [15,16,17]. Tobacco overexpressing MPGase shows greater tolerance to temperature extremes [18].

Increased synthesis of AsA under the control of MPGase is a key response to environmental stress as can be demonstrated in several examples. The MPGase gene *VTC1* may be induced under oxidative stress, increasing the formation of AsA [19], while *Arabidopsis* mutant *vtc1* exhibits increased sensitivity to salt, arising from reduced synthesis of AsA with a corresponding lower capacity for the removal of ROS [20]. The salt tolerance of *Arabidopsis* is increased by enhanced transcription of *VTC1* under the control of factor AtERF98 [21], and the production of AsA in rice root under salt stress is increased via the *OsVTC1-3* MPGase gene, leading to improved growth [16].

Dissecting the key genes involved in rice salt tolerance is an important objective for accelerating rice breeding [22]. To identify putative salinity tolerance genes, a library of ethane methyl sulfonate (EMS)-induced *japonica* rice cv. Nipponbare (NPB) mutants was screened under salinity conditions. We isolated a highly salt stress-sensitive mutant *ss3* and cloned the gene responsible, which encodes MPGase. Genetic, phenotypic, and biochemical analyses revealed that *SS3* modulates AsA synthesis and ROS scavenging in rice.

## 2. Results

### 2.1. Isolation of a Rice Mutant Sensitive to Salt Stress

**Figure 1:**
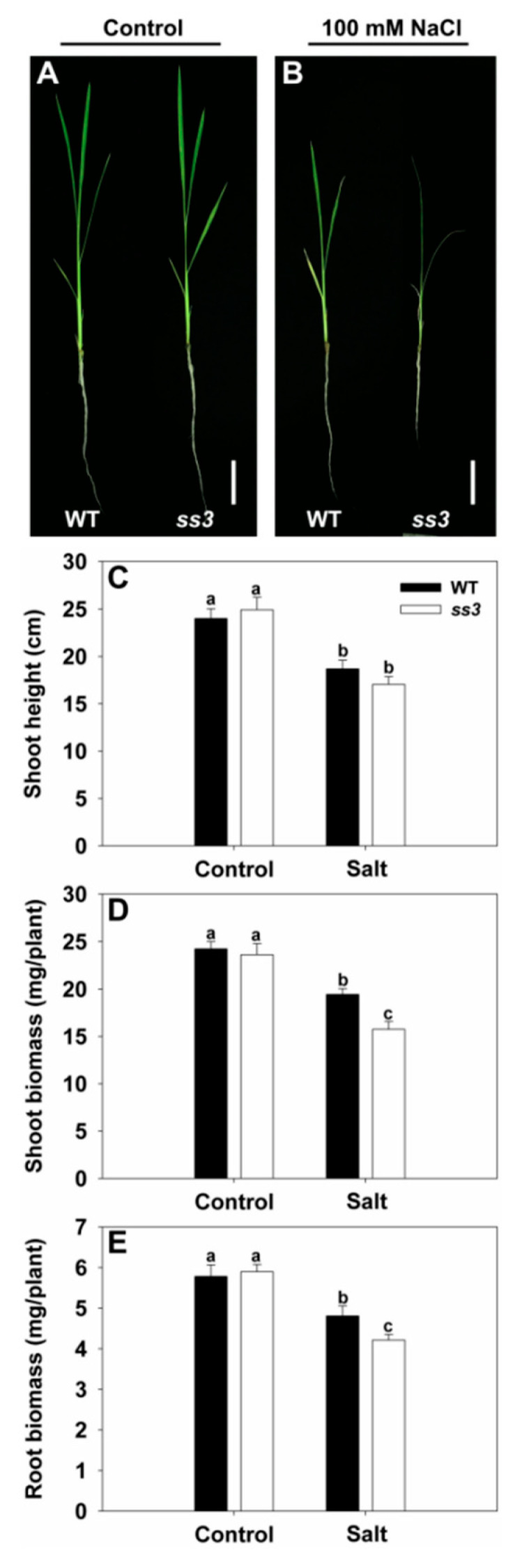
Growth performance in response to salinity stress of *ss3* mutant compared with WT. Growth performance of WT and *ss3* mutants under normal (**A**) or 100 mM NaCl treatment (**B**). Ten-day-old seedlings were treated for four days with or without the addition of 100 mM NaCl. Shoot height (**C**), shoot biomass (**D**), and root biomass (**E**) of the seedlings under normal and salt stress conditions. Bars = 4 cm. The values are means ± SE of five replicates. Significant differences at *p* < 0.05 are indicated with different letters.

A library of EMS-induced *japonica* rice mutants was screened under salinity stress conditions using 100 mM NaCl. We identified a mutant that is highly sensitive to salt stress, designated *ss3*. As shown in Figure 1A,B, no differences were detected between the growth of wild type (WT) and *ss3* under normal conditions. Upon exposure to salinity, *ss3* plants wilted severely and foliar chlorosis was observed, whereas WT showed only slight wilting and withered blade tips (Figure 1A,B). The stress treatment led to suppression of growth in both WT and *ss3,* but there was no significant difference in shoot height between WT and *ss3* (Figure 1C). However, the shoot and root biomasses of *ss3* seedlings were significantly reduced to 81% and 87% of those of WT under equal salt stress, respectively (Figure 1D,E).

### 2.2. Map-Based Cloning of SS3

**Figure 2:**
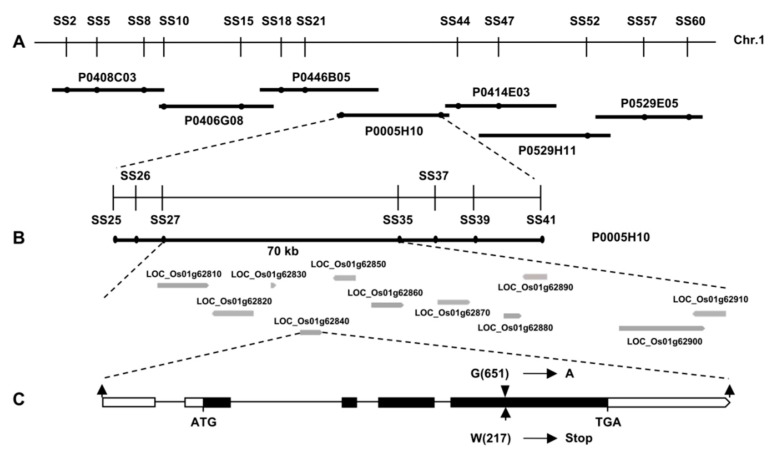
Map-based cloning of *SS3*. (**A**) *SS3* was initially mapped to chromosome 1 (Chr.1) and fine mapping was carried out with markers developed based on the sequence of BAC clone P0005H10. The *SS3* locus was narrowed to a 70 kb genomic DNA region between markers SS27 and SS35. (**B**) Eleven open reading frames were located in the region. (**C**) Gene structure of the *SS3* candidate LOC_Os01g62840. Start codon (ATG) and the stop codon (TGA) are indicated. Blank boxes indicate coding sequence. Arrows show mutation site of *ss3*.

Positional cloning was employed to identify the *SS3* gene and characterize the *ss3* phenotype. The mutant was crossed with *indica* variety TN1 to obtain the F_2_ population for mapping. The *SS3* gene occurs on chromosome 1 between SSR markers SS2 and SS60 (Figure 2A). Fine mapping via additional PCR-based markers localized *SS3* to a 70 kb segment on BAC clone P0005H10 between SS27 and SS35 (Figure 2B). Eleven open reading frames were identified in this interval by database searching (https://ensembl.gramene.org/Oryza_sativa/Location/View?db=core;r=1:36373818–36443332 accessed on 10 June 2021) and the corresponding sequences in the *ss3* and WT parents were compared. The *ss3* genome was found to contain a G to A substitution at position 651 in exon 4 of the predicted MPGase gene LOC_Os01g62840. This results in a change from a tryptophan codon to a stop codon, with truncation of the protein (Figure 2C).

### 2.3. SS3 Is Involved in AsA Production and ROS Scavenging in Rice

**Figure 3:**
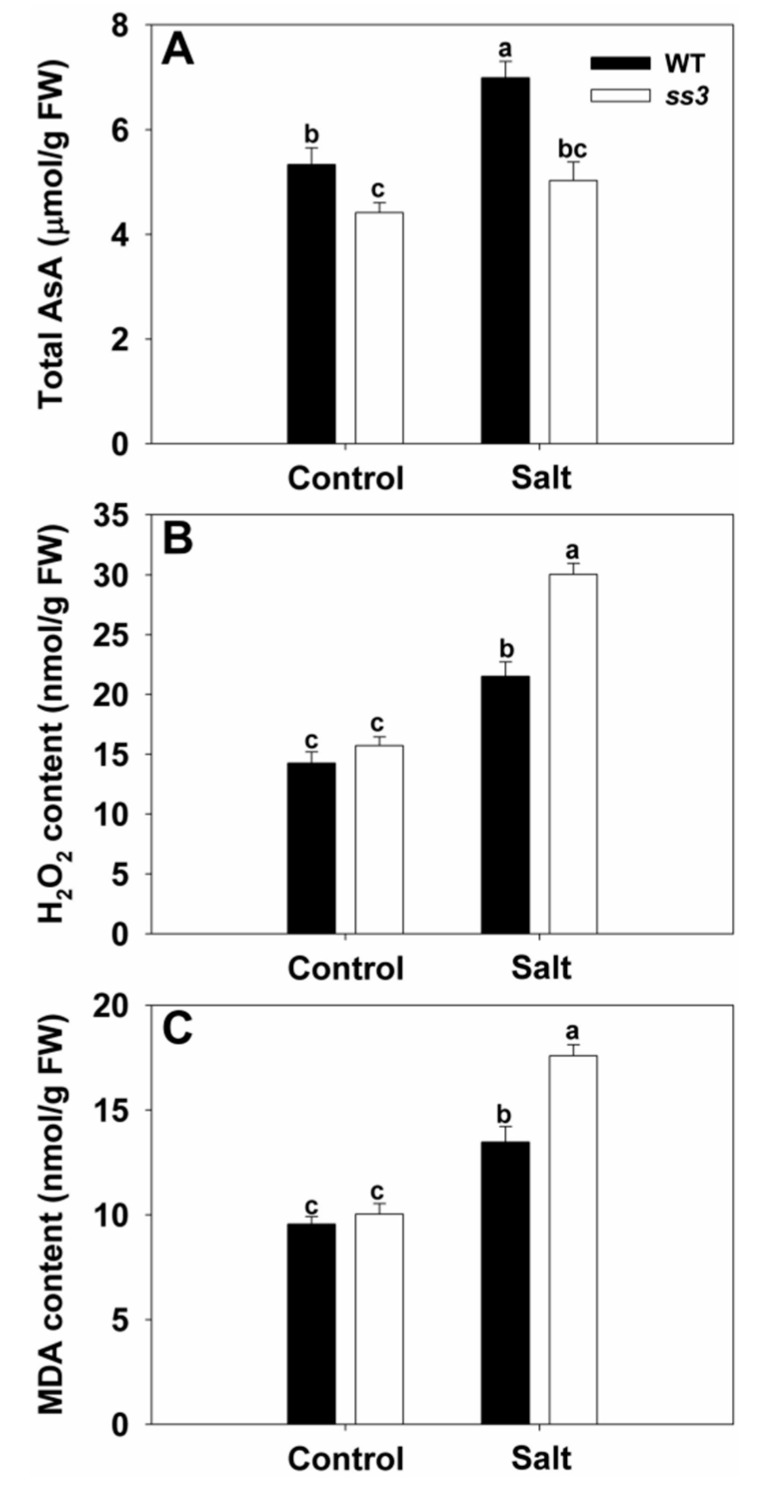
ROS accumulation in response to salinity stress in *ss3* mutant compared with WT. Growth conditions and treatments were the same as described in Figure 1. The AsA content (**A**), H_2_O_2_ content (**B**), and MDA content (**C**) are shown. The values are means ± SE of five replicates. Significant differences at *p* < 0.05 are indicated with different letters. FW—fresh weight.

Since MPGase catalyzes the formation of GDP-D-mannose, the precursor of AsA [23], we determined the AsA contents in WT and *ss3* mutants grown normally or under salt stress. Under control conditions, AsA in the leaves of *ss3* was significantly lower compared to WT, while NaCl treatment markedly increased AsA in WT but not in *ss3* (Figure 3A). Since AsA plays an important role in ROS scavenging to protect rice against oxidant-induced damage under environmental stress [15,16], we also monitored H_2_O_2_ and MDA. WT and *ss3* had similar levels of both under normal conditions, but when grown with added NaCl, the H_2_O_2_ and MDA contents of *ss3* were 40% and 31% higher than those of WT, respectively (Figure 3B,C), indicating that functional *SS3* is important for ROS scavenging in rice under salinity conditions.

**Figure 4:**
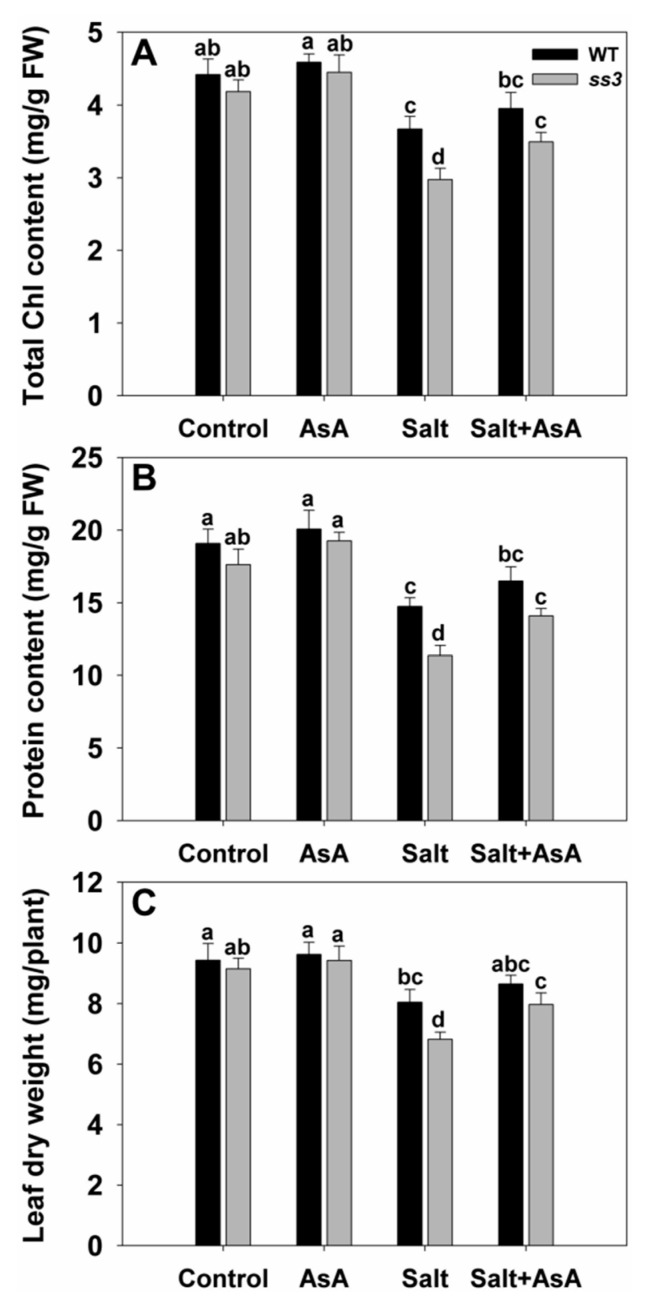
Exogenous AsA mitigated the salt hypersensitivity of *ss3* mutants. Ten-day-old seedlings were transferred for four days to a nutrient solution containing 0 mM or 100 mM NaCl supplied with or without 1 mM AsA. The chlorophyll (Chl) content (**A**), protein content (**B**), and leaf dry weight (**C**) were assayed. The values are means ± SE of five replicates. Significant differences at *p* < 0.05 are indicated with different letters. FW—fresh weight.

Further, we found that adding exogenous AsA restored the tolerance of *ss3* seedlings to salinity. Physiological indicators such as chlorophyll content, protein content, and leaf dry weight of *ss3* were then similar to that of WT (Figure 4). Thus, *SS3* mediates the response of rice to salt stress through the regulation of AsA biosynthesis.

### 2.4. Generation of Complementation Lines for SS3

**Figure 5:**
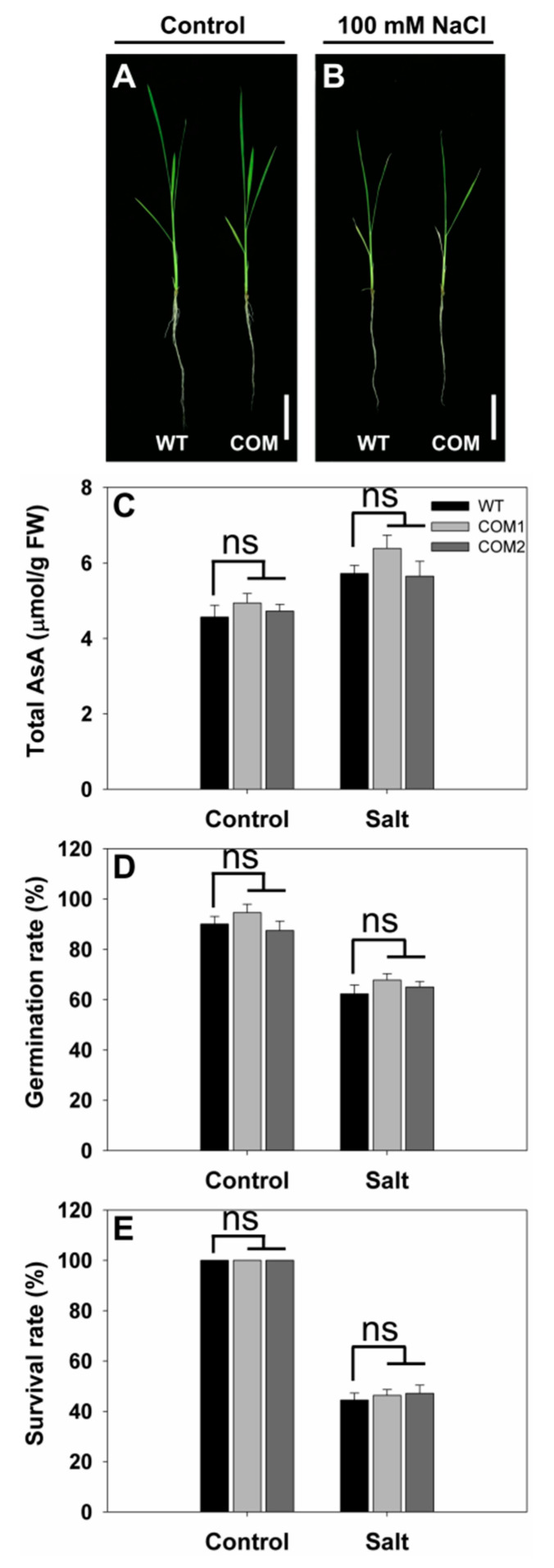
Growth performance in response to salinity stress of complementation lines compared with WT. Growth conditions and treatments were the same as given in Figure 1. Growth performance of WT and the complementation lines under normal (**A**) or 100 mM NaCl treatment (**B**). Bars = 5 cm. (**C**–**E**), AsA content (**C**), germination rate (**D**), and survival rate (**E**) of the seedlings under normal and salt stress. The values are means ± SE of five replicates. FW—fresh weight; ns indicates non-significant differences.

To further confirm that the loss of function of *SS3* was responsible for the salt-sensitive phenotype, we generated two complementation lines (COM1 and COM2) for *SS3*. These lines rescued the salt-hypersensitive phenotype of *ss3* mutants (Figure 5A,B). As shown in Figure 5C, the AsA contents of leaves of the complementation lines were restored to WT levels. Under normal and salt stress conditions, there were no obvious differences in the germination rate and survival rate between WT and complementation lines (Figure 5D,E). These results confirm that disruption of *SS3* results in hypersensitivity to salinity stress.

### 2.5. SS3 Is Induced by Salinity Stress

**Figure 6:**
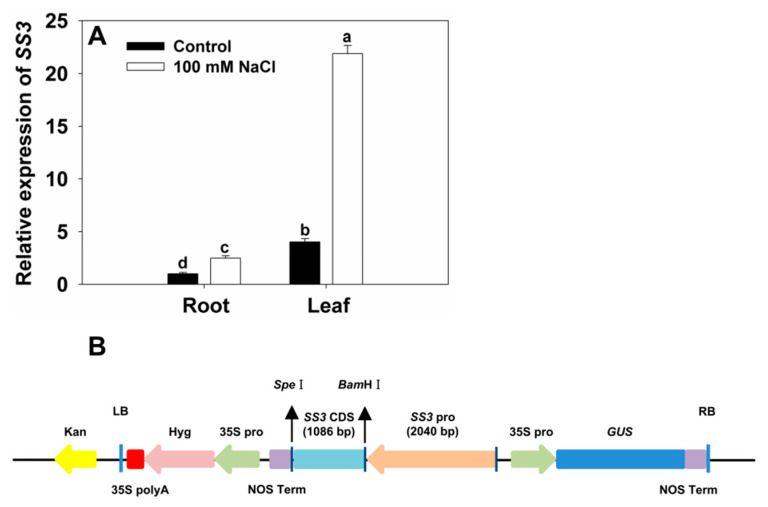
Effect of salt stress on the expression of *SS3*, and construction map of *SS3p:SS3* vector. (**A**) Relative expression levels of the *SS3* gene in rice roots and leaves under normal and salinity stress. The abundance of *SS3* transcript in the non-stressed roots was normalized to 1. The values are means ± SE of three replicates. Significant differences at *p* < 0.05 are indicated with different letters. (**B**) Construction map of *SS3p:SS3* expression vector.

To further assess the role of *SS3* in the plant response to salt stress, we tested whether salinity could induce *SS3* expression. We grew 10-day-old WT NPB rice plants with the addition of 100 mM NaCl and investigated the expression patterns of *SS3* in tissues including roots and leaves using qRT-PCR. *SS3* transcript levels increased 1.5- and 4.4-fold in NaCl-treated roots and leaves, respectively, compared with the untreated controls (Figure 6A).

### 2.6. Generation of Transgenic Rice Expressing SS3p:SS3

To further determine the relationship between *SS3* regulation of AsA synthesis and salt sensitivity, we manipulated the expression of *SS3* gene by transforming its native promoter *SS3p*:*SS3* construct into rice (Figure 6B).

We obtained 15 independent *SS3p:SS3* transgenic lines in the T_0_ generation as demonstrated by Southern blot analysis. Four null segregants were detected in the T_1_ generation by GUS staining, which were used in the phenotype analysis. Since no significant difference in salt sensitivity was observed between WT and the null segregants in the T_1_ generation, as determined by a comparison of the growth characteristics and chlorosis of leaves, we used WT as the single negative control in hydroponic experiments on the transgenic lines in the T_2_ generation. In addition, GUS staining was performed on each single-copy transgenic line of the T_2_ generation, and two homozygous lines (L1 and L2) were selected for further detailed analysis.

### 2.7. Effects of SS3p:SS3 Expression on Rice Growth and Na^+^/K^+^ Homeostasis under Salt Stress

**Figure 7:**
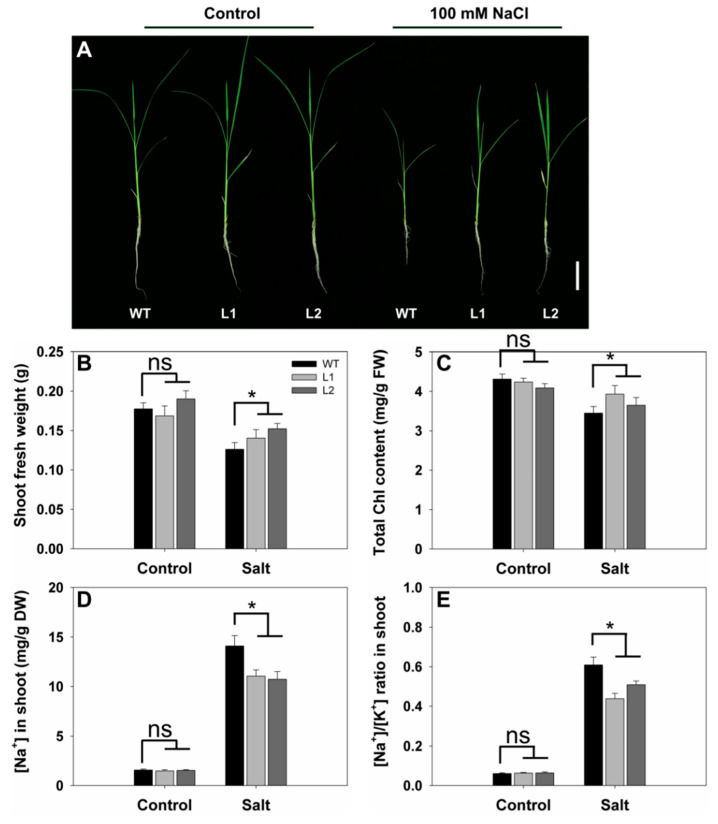
Growth performance in response to salinity stress of *SS3p:SS3* transgenic lines compared with WT. Ten-day-old seedlings were treated for six days with or without addition of 100 mM NaCl. (**A**) Growth performance of WT and *SS3p:SS3* transgenic lines under normal and 100 mM NaCl treatment. Bars = 4 cm. Shoot fresh weight (**B**), chlorophyll (Chl) content (**C**), Na^+^ concentration (**D**), and Na^+^/K^+^ ratio (**E**) in shoots of the seedlings under normal and salt stress. The values are means ± SE of five replicates. Significant differences between WT and transgenic lines at *p* < 0.05 are indicated with asterisks; ns denotes non-significant differences. FW—fresh weight. DW—dry weight.

As shown in Figure 7A, no morphological differences were observed between WT and the transgenic lines grown without added salt. Under salt stress, the growth of WT was inhibited to a greater extent than that of the transgenic lines and was accompanied by more severe wilting in the leaves (Figure 7A). To quantify the phenotypes, we compared the shoot fresh weight and chlorophyll contents of the WT and transgenic lines under salt stress. The magnitude of the reduction in shoot growth of *SS3p:SS3* transgenic plants was significantly less than that found in WT. When grown in salt solution, the shoot fresh weight of the transgenic lines was approximately 16% higher than that of WT (Figure 7B) and chlorophyll contents were also higher in transgenic lines compared with those in WT plants (Figure 7C). Thus, the *SS3p:SS3* transgenic lines were demonstrably more tolerant to salinity stress than WT.

We determined the Na^+^ and K^+^ contents in the shoots of the WT and transgenic lines. Under control conditions, we observed no substantial differences in either Na^+^ concentration or Na^+^/K^+^ ratios between the plant materials (Figure 7D,E). Six days after treatment with an added 100 mM NaCl, transgenic plants accumulated less Na^+^ in shoots than WT plants (Figure 7D). Furthermore, the Na^+^/K^+^ ratios in the shoots of the transgenic lines were 17–28% lower than the corresponding values for WT (Figure 7E).

### 2.8. SS3p:SS3 Transgenic Plants under Salt Stress Present Altered Expression of Senescence- and Autophagy-Related Genes

**Figure 8:**
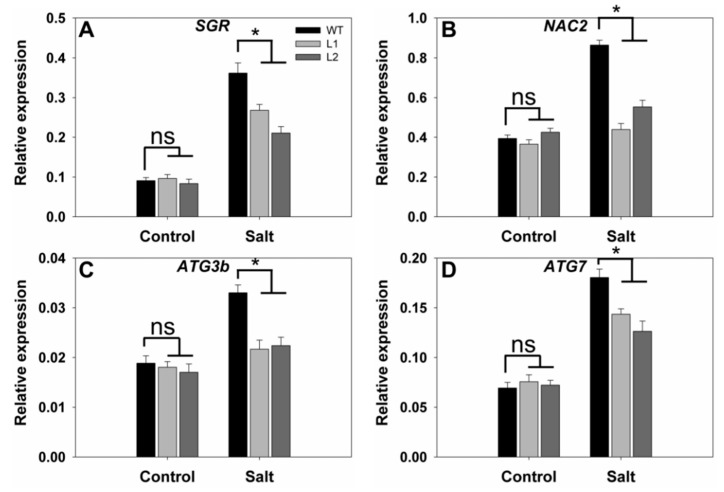
Expression levels in response to salinity stress of senescence- and autophagy-related genes in *SS3p:SS3* transgenic lines compared with WT. Growth conditions and treatments were as described in Figure 7. RNA was extracted from the leaf blades and qRT-PCR was used to detect transcript levels of *OsSGR* (**A**), *OsNAC2* (**B**), *OsATG3b* (**C**), and *OsATG7* (**D**). The values are means ± SE of three replicates. Significant differences between WT and transgenic lines at *p* < 0.05 are indicated with asterisks; ns indicates non-significant differences.

Premature leaf senescence is an important response of plants to various external pressures such as salinity stress [24]. In order to further confirm that *SS3* positively regulates the tolerance of rice to salt stress, we performed gene expression profiling of transgenic lines for comparison with WT under normal and salinity conditions. Under normal growth conditions, we observed relatively weak expression and insignificant variations in transcriptional levels for the tested genes in both WT and transgenic plants (Figure 8). The presence of NaCl up-regulated all of the tested genes, although the extent of the induction was less for the transgenic lines than for WT. Expression levels of *OsSGR*, *OsNAC2*, *OsATG3b,* and *OsATG7* in the leaves of transgenic lines were 59–74%, 51–64%, 65–68%, and 70–80% of those in WT leaves, respectively (Figure 8). These results indicate that expression of *SS3p:SS3* additionally enhances salt tolerance in rice by alleviating leaf senescence induced by salinity stress.

## 3. Discussion

The biosynthetic pathway for ascorbic acid involves a sequence of key enzymes, with MPGase as the first rate-limiting step [25,26]. MPGase has a critical role in the antioxidant response of seedlings to salt stress [23,27]. Many studies have reported that, in plants, changes in the MPGase enzyme can dramatically influence AsA synthesis, modifying tolerance to salt stress [21,28]. In *Arabidopsis*, transcription factor AtERF98 activates the expression of *VTC1*, enhancing the production of AsA and improving tolerance [21], while conversely, a point mutation in *VTC1* had a severe impact on seedling susceptibility [20]. In contrast, the COP9 complex subunit CSN5B interacts with VTC1 to promote its degradation, but plants bearing a point mutation of *CSN5B* showed reduced degradation of VTC1, higher levels of AsA, and enhanced tolerance to salt [27].

Other VTC pathway genes play similar roles in the impact of salt stress on seedling growth. Abscisic acid pathway transcription factor ABI4 binds to the promoter of *VTC2,* reducing the production of the protein and thus the production of AsA during early plant growth, with *Arabidopsis* seedlings becoming less tolerant to salt [17]. The same effects are found in rice. Transcription of *OsVTC1-3* increased under salt stress, while its inhibition resulted in stunting of growth [16]. Again, expression of *OsMPG1* increases when rice is grown in saline conditions, while its overexpression in tobacco was found to reduce salt stress [23].

Our results demonstrate that *SS3* is up-regulated in seedlings challenged by an increase in salinity (Figure 6). In addition, plasmid *SS3p:SS3* expression resulted in improved rice shoot growth and assisted in the maintenance of Na^+^/K^+^ ratios in challenged plants. In contrast, *ss3* mutants grew less successfully than WT rice under similar salinity conditions (Figure 1 and Figure 7). MPGase SS3 is essential for the normal growth of rice seedlings cultivated under salt stress. The reduction in AsA levels in the *ss3* mutant explains its greater susceptibility to salt. Our complementation line experiments show (Figure 3A and Figure 5C) that returning AsA levels in the mutant to those of WT rice restored growth to that found in WT, as did the addition of exogenous AsA (Figure 4).

Damage to plants caused by salt is partly due to the induction of ROS [29]. Increased ROS levels could be a significant factor in the greater severity of leaf senescence in *ss3* seedlings compared to WT under identical salt stress conditions. Excessive ROS levels disrupt cell membranes and degrade cell components, leading to premature leaf senescence [30,31,32]. One major consequence of senescence is the loss of chlorophyll [24,33], as was observed for stressed *ss3* seedlings relative to WT (Figure 4A). In contrast, salt-tolerant transgenic *SS3p:SS3* plants had raised chlorophyll levels (Figure 7C). Loss of protein is another major indicator of senescence [34], and *ss3* seedlings under stress contained significantly less protein (Figure 4B). Membrane disruption during senescence may arise via lipid peroxidation [31], which gives rise to MDA as a significant product [24], and increased levels were found in leaves from stressed *ss3* seedlings (Figure 3C). In addition, elevated levels of the ROS component H_2_O_2_ were found in the leaves of stressed mutants relative to WT (Figure 3B).

Senescence is a dynamic process associated with large-scale transformations in overall gene expression and characteristic increases in the products of a set of senescence-associated genes including *OsSGR* [31,34]. Similarly, the senescence-related transcription factors *OsNAC2*, *OsWRKY23,* and *OsWRKY72* were all up-regulated [24,35]. Following salt treatment, we found that both transcription of *OsSGR* (Figure 8A) and expression of *OsNAC2* (Figure 8B) were lower in *SS3p:SS3* lines than in WT.

Autophagy is another significant process during leaf senescence [24] and three autophagy-related genes (*OsATG4b*, *OsATG8a,* and *OsATG18b*) were found to show increased expression in rice [34]. In our study, the mRNAs of two other autophagy-related genes (*OsATG3b* and *OsATG7*) were less abundant in *SS3p:SS3* lines than WT when grown under salt challenge (Figure 8C,D). Expression of these genes (Figure 7A) was also in agreement with the superior performance of *SS3p:SS3* lines relative to WT under salt stress.

Neutralization of ROS is critical for plants to adapt to difficult conditions [7,15], and MPGase has been identified as important for ROS scavenging in tobacco and *Arabidopsis*, both dicots [20,23]. The ability of *Arabidopsis* to deactivate ROS was compromised by mutations of *VTC1*. This reduced its ability to withstand ozone and other oxidants along with its tolerance to salinity [17,20]. Similarly, under saline conditions, inhibition of OsVTC1-3 led to a rapid increase in hydrogen peroxide concentration in rice roots [16]. In plants under salt stress, AsA could function as a primary antioxidant, i.e., plants react directly with AsA, and may also remove ROS via the AsA-GSH cycle [15,36]. Our results clearly show that SS3 increases the resistance of rice to saline conditions by control of AsA synthesis and the concomitant removal of ROS.

## 4. Materials and Methods

### 4.1. Plant Materials and Growth Conditions

Rice seeds (*Oryza sativa* ssp. *japonica* cv. NPB) from a library of EMS-induced mutants were surface-sterilized and germinated. Plants were cultured hydroponically in a growth chamber under a 14 h photoperiod at 30 °C during the light period and 25 °C during the dark period. The hydroponic solution was replaced every two days. The relative humidity was maintained at ~70%.

To screen for salinity stress-responsive mutants and to investigate the effect of high salinity on early seedling growth of WT and complementation lines, uniformly sized 10-day-old seedlings were treated for four days with a nutrient solution [37] containing a final concentration of 100 mM NaCl. The growth performance and the extent of chlorotic damage were determined [1]. To determine the effect of salt stress on the germination rate, rice seeds were sterilized and grown at 30 °C for seven days on 1/2 strength Murashige and Skoog (MS) medium (3% sucrose, 1% agar, pH 5.8) supplemented with or without 100 mM NaCl under a 12 h light/12 h dark photoperiod (100 µmol m^−2^ s^−1^ light intensity). The impact of salt stress on the survival rate of WT and complementation lines was assessed by growing them on normal 1/2 MS solid medium for two weeks, followed by irrigation with or without 150 mM NaCl for seven days. After three days of recovery, the survival rates were recorded; seedlings with green leaves were regarded as having survived. The survival rate was the ratio of surviving seedlings to the total number, as previously described [38].

To assess the effect of exogenous AsA on seedling growth and salt tolerance, 10-day-old seedlings were transferred for four days to a nutrient solution containing 0 mM or 100 mM NaCl supplemented with or without 1 mM AsA, as described by Wang et al. [36]. To investigate the differences between *SS3p:SS3* transgenic lines and WT in response to salinity stress, 10-day-old seedlings were treated for six days with or without 100 mM NaCl. Shoots, roots, or leaf blades of the seedlings were collected after treatments, and physiological parameters and gene expression patterns were examined. Experiments were performed in triplicate with 5 individuals per genotype for each treatment.

### 4.2. Map-Based Cloning

For map-based cloning, the mutant (female parent) in the *japonica* background was hybridized with the *indica* cultivar TN1 to construct the segregating population. Localization was initially determined with simple sequence repeat (SSR) markers covering all 12 chromosomes [39]. For further mapping, new markers between the two flanking markers were designed based on the differences between the genomic DNA sequences of *japonica* variety NPB and *indica* variety 9311. All of the PCR products were separated on 5% agarose gels for visualization. Primers used for mapping the *SS3* gene are listed in Table S1. The methods used for marker design and map-based cloning were as described by Yang et al. [39].

### 4.3. Generation of Transgenic Lines

For complementation of the *ss3* mutation, we cloned a 4807 bp genomic DNA fragment containing the *SS3* coding region into the binary vector pCAMBIA1300 along with the upstream and downstream sequences. The recombinant vector was introduced into the calli generated from mature seed embryos of the *ss3* mutant by the *Agrobacterium*-mediated method [40].

To construct the *SS3p:SS3* vector, the 1086 bp coding sequence (CDS) of *SS3* was amplified with NPB cDNA as a template. After purification, it was connected to the pTCK303 linear vector, which was obtained from pTCK303 digested by *BamH*I and *Spe*I, using the GBclonart seamless cloning kit (Genebank Biosciences Inc., Zhangjiagang, China), and the intermediate pTCK303-SS3 vector was obtained. The *SS3* promoter (2040 bp upstream of the initial codon) was amplified with NPB DNA as the template, and then purified and ligated to the pTCK303-SS3 linear vector, which was obtained from pTCK303-SS3 digested by *BamH*I, and the final construct was transformed into NPB as described above.

### 4.4. Determination of AsA Content

Rice seedling leaves were harvested and stored in liquid nitrogen. An aliquot of 0.2 g (fresh weight) was ground to a fine powder in liquid nitrogen. The extraction and measurement of AsA were performed as described by Wang et al. [27].

### 4.5. Determination of H_2_O_2_ and MDA Content

Seedling leaves harvested after salt treatment were ground and stored at −80 °C. The H_2_O_2_ content was assessed according to Chen et al. [41], while MDA was determined following Chen et al. [42].

### 4.6. Measurement of Chlorophyll and Soluble Protein Content

Chlorophyll content was determined by the method of Chen et al. [42], and soluble protein was quantified as described by Chen et al. [24].

### 4.7. Determination of Sodium and Potassium Ions

Shoots were excised from intact seedlings and dried in air to a constant weight at 70 °C. After digestion with nitric acid at 100 °C, Na^+^ and K^+^ were measured with an Optima 2100DV ICP emission spectrometer (Perkin Elmer Inc., Shelton, CT, USA) using the method of Chen et al. [43].

### 4.8. Quantitative Real Time PCR (qRT-PCR)

qRT-PCR was carried out according to Chen et al. [44]. RNA was extracted from the roots and leaf blades of seedlings grown under both normal and salt stress conditions. The rice gene *UBQ5* (LOC_Os01g22490) was chosen as the reference sequence, and relative transcript abundances were calculated as given by Chen et al. [45]. The sequences of all primers used in the qRT-PCR experiments are listed in Table S2.

### 4.9. Statistical Analysis

Analyses of variance were carried out using SPSS v10 software (SPSS Inc., Chicago, IL, USA). Statistically significant differences between the performance of the various genotypes and WT, or between different treatments (as determined by Tukey’s test), are denoted by the inclusion on the histograms of a specific letter or an asterisk.

## 5. Conclusions

A salt-hypersensitive mutant was obtained by screening an EMS mutant library under salinity stress, and gene *SS3* was identified. We cloned the *SS3* gene and demonstrated that it plays a novel role in response to salinity stress, i.e., rice can specifically activate the transcription of *SS3* to promote AsA synthesis, thus scavenging ROS in vivo to improve salt tolerance. Further investigations will focus on the *SS3*-mediated stress signaling pathway in rice to provide both genetic resources and a theoretical basis for enhancing salinity resistance in crops.

## Data Availability

Data are contained within the article and Supplementary Material.

## Supplementary Materials

The following supporting information can be downloaded at: https://www.mdpi.com/article/10.3390/ijms231810338/s1.
