# Supplementary material for: Salt Tolerance of Rice Is Enhanced by the SS3 Gene, Which Regulates Ascorbic Acid Synthesis and ROS Scavenging
Source: Int J Mol Sci. 2022 Sep 7;23(18):10338. doi: 10.3390/ijms231810338 (PMC9499165; doi:10.3390/ijms231810338)
Supplement: Supplementary file 1 [file ijms-23-10338-s001.zip › ijms-1892271-supplementary.pdf]

Supplementary Information

Table S1. Marked primer used for gene mapping.

Marker	Forward primer (5'—3')	Reverse primer (5'—3')
SS2	TATAGTCGAACGTCCCTCTGA	ATCTACTCCGTACATACTTTTGC
SS5	ATTGGAGATTGGCCTGTTGA	CGAGTGGTAAAGCCGTTTGT
SS8	AAAGAAATTCAACTTCGTCA	AACTTCCACTATCACAGCAT
SS10	TGTAAGTCATTCTAGCATTTCTC	ATGTTGCCTTCTACTCCCTC
SS15	GGAAAAGAATCCTGCAATGGAG	CGTGTCCTCCTCCGGTAGAAG
SS18	TATTTGGACAGCACCTTTTA	ATGCTTTGATGATACTCCCT
SS21	ATCAGTGGCTAGATCAAAAG	GAACCTAAATTAACCAAAGT
SS25	TCTTGCTACCATACCCTCTTT	GGAACGCTTATCCAGTTTCT
SS26	GACGATTGATCTATCTCATTAC	GGCGACGAGTACATAGCATG
SS27	CCCAGGGGCTTCATGCTCGC	CTCCCGCATCGGCAAGGCAC
SS35	ATTATTCCTATTTACAGGTTCT	TACTAGCATTGACATGGCAC
SS37	GGCACATTCTATTACTATGA	CAAGTTCTACAGTACCTCGC
SS39	TTAAATAGAATTGAAATCCTCC	ATACACTTCGCGAGATGTCC
SS41	CGCACGGCGCCAGCTTAAGC	TGGTTTCTCCTGGTGGTTGC
SS44	CATAACCCAATATTATCATATC	GATAACTTATTCCGTCCATA
SS47	GGCTTTCACGACAAGGCATT	TGGATTCAACGGGGTGGTGT
SS52	TAATGGTATGTATCTAATTAACCC	GCTTCATTTAGATGGAGCTT
SS57	ACGGAGTCACGGCCACCCTC	ACAAGCGGCAGCGCCCAATG
SS60	ACTGTGAAATCCGGTTCTTT	ATCAAGTTTGTATGGGGAGA

Table S2. Primer sequences used for qRT-PCR assays.

Gene	Primer ID	Primer sequences
UBQ5	F(5'-3')	CTCGCCGACTACAACATCCA
	R(5'-3')	TCTTGGGCTTGGTGTACGTCTT
SS3	F(5'-3')	GTCATGTGAACTAACCCTCC
	R(5'-3')	GAGTTTCTTCTGGTCCTCTTG
OsSGR	F(5'-3')	GCAATGTCGCCAAATGACG
	R(5'-3')	GCTCACCACACTCATTCCCTAAAG
OsNAC2	F(5'-3')	AAAAACAACCGCATTGGCAG
	R(5'-3')	AGTCCTCATCTCCTCTGTCTAATCC
OsATG3b	F(5'-3')	CGAGCAAGAGGAAGCCGTAT
	R(5'-3')	CGTCTTACGCAAGGCACGTT
OsATG7	F(5'-3')	GTGATGCAGGTGATCAACGAA
	R(5'-3')	GCCGCTGATTTCATCAAGTCA
